# Laser-Cutted Epidermal Microfluidic Patch with Capillary Bursting Valves for Chronological Capture, Storage, and Colorimetric Sensing of Sweat

**DOI:** 10.3390/bios13030372

**Published:** 2023-03-12

**Authors:** Yuxin He, Lei Wei, Wenjie Xu, Huaping Wu, Aiping Liu

**Affiliations:** 1 Key Laboratory of Optical Field Manipulation of Zhejiang Province, Zhejiang Sci-Tech University, Hangzhou 310018, China; 2 School of Physics and Electronics Engineering, Fuyang Normal University, Fuyang 236037, China; 3 Key Laboratory of Special Purpose Equipment and Advanced Processing Technology, Ministry of Education and Zhejiang Province, College of Mechanical Engineering, Zhejiang University of Technology, Hangzhou 310023, China

**Keywords:** laser-cutting technology, microfluidics, capillary bursting valves, glucose level, colorimetric sensing

## Abstract

Flexible wearable microfluidic devices show great feasibility and potential development in the collection and analysis of sweat due to their convenience and non-invasive characteristics in health-level feedback and disease prediction. However, the traditional production process of microfluidic patches relies on resource-intensive laboratory and high-cost facilities. In this paper, a low-cost laser-cutting technology is proposed to fabricate epidermal microfluidic patches for the collection, storage and colorimetric analysis of sweat. Two different types of capillary bursting valves are designed and integrated into microchannel layers to produce two-stage bursting pressure for the reliable routing of sweat into microreservoirs in sequential fashion, avoiding the mixing of old and new sweat. Additionally, an enzyme-based reagent is embedded into the microreservoirs to quantify the glucose level in sweat by using colorimetric methods, demonstrating a high detection sensitivity at the glucose concentration from 0.1 mM to 1 mM in sweat and an excellent anti-interference performance that prevents interference from substances probably existent in sweat. In vitro and on-body experiments demonstrate the validity of the low-cost, laser-cut epidermal microfluidic patch for the chronological analysis of sweat glucose concentration and its potential application in the monitoring of human physiological information.

## 1. Introduction

Nowadays, blood examination is widely used in clinical practice due to its advantage of high detection accuracy. At the same time, because of its puncturing characteristics, professional persons and specialized collection/analysis instruments are necessary. Patients who need a long-term blood collection go through both psychological and physiological stress during repetitive puncturing. Meanwhile, a large number of biomarkers, such as glucose, lactate, urea, sodium, potassium and proteins, also exist in human sweat [1,2]. Compared with blood collection, sweat collection is more convenient and non-invasive; thus, it is expected to be an ideal method for health information monitoring, especially for patients with diabetes with daily sampling requirements [3]. Many studies have found that the concentrations of biomarkers in sweat are highly correlated with those in blood. Although the change in sweat glucose concentration has a short lag compared with that in blood glucose concentration, sweat glucose can still reflect the health status and the change trend of human blood glucose [4]. It is hoped that sweat sensing can be introduced into home medical treatments to achieve the early warning and diagnosis of diseases. Recently, many methods have been proposed for sweat collection to obtain meaningful information about the physiological state of the body [5,6]. Thereinto, microfluidic chips integrated with microsensors are the ideal platforms to perform the monitoring of individual health conditions based on sweat analyses, where sweat can be collected, stored and analyzed in situ [7]. Sweat glands naturally secrete sweat after an increase in body heat or a rise in ambient temperature, and the osmotic pressure between plasma and the epidermis pushes the secreted sweat toward the skin surface, where it enters the analysis area of microfluidic chips [8,9]. However, there is an inevitable mixing between measured sweat and that to be measured on the skin surface, which greatly affects the measured accuracy of biomarkers due to biomarkers’ metamorphosis with time [10]. In order to avoid this problem, the chronological collection strategy based on microfluidic patches has been proposed as an effective way to realize the precise control of sweat collection, storage, in situ detection and outflow along a well-designed path under the assistance of multifunctional valves [11]. For example, Choi et al. introduced an approach for guiding sweat flow in a sequential manner in a skin-mounted microfluidic device via carefully designed capillary bursting valves (CBVs) [12]. Kim et al. used a valving technology to control the flow of sweat from an inlet to an isolated reservoir in a well-defined sequence [13]. Kim et al. fabricated a microfluidic system with microchannels, reservoirs, valves and other components in low-modulus elastomers [14]. However, these elaborate microchannels and valves integrated into microfluidic patches are usually well prepared by using soft-lithography technology via silicon molds, and the entire production process relies on resource-intensive laboratory and high-cost facilities [15]. Therefore, a flexible microfluidic patch with an ingenious design and a simplified preparation method is both imperative and challenging for convenient sweat collection and sensing for patients with diabetes with daily sampling requirements.

**Figure 1:**
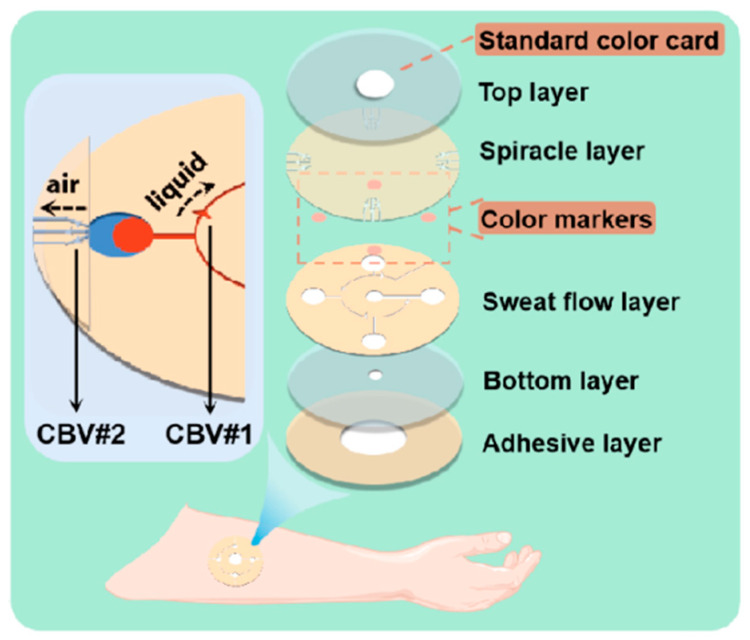
Enlarged view of microfluidic patch with capillary bursting valves (CBVs).

In this study, we fabricate a microfluidic patch by utilizing a low-cost, simple laser-cutting manufacturing technique using double-sided tape and transparent polyimide (PI) films. CBVs with an adjustable bursting pressure (BP) are also designed and achieved within microchannels by using this laser-cutting method for the chronological capture and storage of sweat to avoid the mixing of the sweat tested and that to be tested (Figure 1). Compared with conventional photolithography, the laser-cutting method just needs one step to make programmable microchannels and CBVs without predesigned templates, and it does not need an ultra-clean room or other expensive experimental conditions. Moreover, the glucose concentration in sweat detected in chronological order makes it possible to monitor glycemic elevation in a timely manner. The color markers in the microreservoir of the microfluidic patch have a sensitive color reaction to glucose at different concentrations, presenting a high sensitivity at the glucose concentration from 0.1 mM to 1 mM in sweat and an excellent anti-interference performance regarding alcohol, urea, chloride and lactic acid, which are probably existent in sweat; this is carried out by contrasting the R value in the RGB color model with a standard one. This indicates the application potential of the proposed microfluidic patch in the field of sweat management and the physiological information monitoring of humans.

## 2. Materials and Methods

### 2.1. Design of Microfluidic Patch

**Figure 2:**
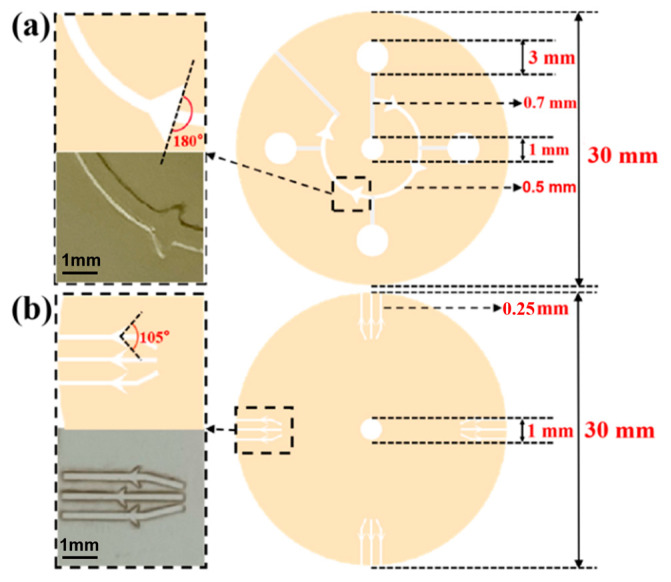
(**a**) The design drawing and size of sweat flow layer with CBV#1. The angle of CBV#1 at inlet is 180. The inlet of CBV#1 is a curved microchannel with 500 μm width and 450 μm height. (**b**) The design drawing and size of spiracle layer with CBV#2. The angle of CBV#2 at inlet is 105°. The inlet of CBV#2 is a rectangular microchannel with 250 μm width and 50 μm height.

**Figure 3:**
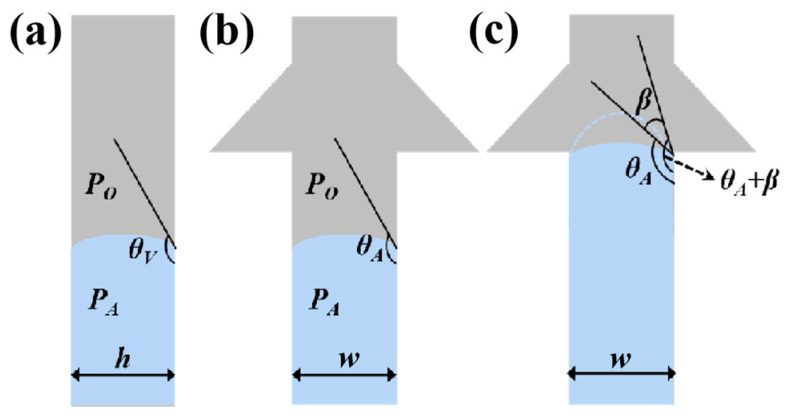
Schematic diagrams of (**a**) side view and (**b**) front view of the valves when liquid is stationary in the microchannel. (**c**) Schematic diagram of the valves when liquid flows into the inlet of CBV#1, with the contact angle increasing from θ_A_ to θ_A+β_.

The whole microfluidic patch consists of six layers and has four microchambers for sweat storage (Figure 1). Two different types of CBVs are designed on two microchannel layers (namely, the sweat flow layer and the spiracle layer) to achieve chronological collection. CBV#1 in the sweat flow layer is proposed to provide the first-stage BP to control the flow and collection of liquid in the four microreservoirs in sequence (Figure 1 and Figure 2a). The second-stage BP is provided by CBV#2 in the spiracle layer. Its function is to balance the pressure inside and outside of the microreservoirs to ensure that, when liquid flows in, the air in the microreservoirs can be moved out smoothly, and the liquid is retained in the microreservoirs without leakage (Figure 2b). When the secreted sweat enters the patch from the inlet (Figure 1), the microchannels can transfer the sweat from the skin surface to the microreservoirs with parallel CBV#2 to release air. When the liquid is stationary in the microchannel, the additional pressure from the difference between the inside and outside flexure liquid levels satisfies Young’s equation (Figure 3a,b) [16]:

(1) $${\Delta P}_{e}=P_{A}-P_{O}=-2\sigma \left[\frac{\cos\theta_{A}}{w}+\frac{\cos\theta_{V}}{h}\right]$$

where *P*_A_ is the pressure from the inside flexure liquid level, *P*_O_ is the pressure from the outside flexure liquid level, and *σ* is the coefficient of the surface tension [17]. *θ*_A_ and *θ*_V_ represent the contact angle of the liquid with the side wall and the top/bottom walls, respectively. *w* and *h* are the width and the height of the microchannel, respectively. As the liquid flows into CBV#1, the width of the microchannel spreads out suddenly, and the three-phase contact line of the liquid stops immediately [18]. Until the contact angle increases from *θ*_A_ to *θ*_A+β_ (Figure 3c), the liquid can enter through CBV#1 [19]. The BP of the liquid at the entrance is [16]

(2) $${\Delta P}_{\mathrm{rec}}=P_{A}-P_{O}=-2\sigma \left[\frac{\cos\theta_{I}}{w}+\frac{\cos\theta_{V}}{h}\right],\;\theta_{I}=\min\left\{\theta_{A+\beta },180\right\},$$

Thus, the liquid flow pressure increases at CBV#1. The expectation of shunting in different branches can be achieved. Additionally, because CBV#2 has a significantly smaller size in height and width than CBV#1 (Figure 2), the BP of the former is much larger than that of the latter, which makes sweat burst out of CBV#1 and advance to the next microreservoir instead of leaking out from CBV#2.

### 2.2. Preparation of Microfluidic Patch

**Figure 4:**
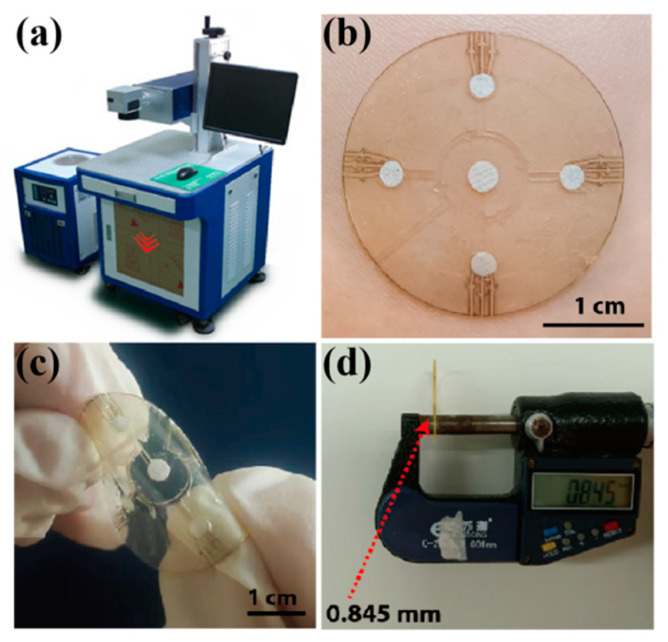
(**a**) The laser-cutting system for microfluidic patch preparation. (**b**) A physical picture of the microfluidic patch on a volunteer’s forearm. The patch is flexible (**c**) and thin, with a thickness of about 0.85 mm (**d**).

For the typical preparation of the microfluidic patch, the spiracle layer with CBV#2 was first fabricated by cutting a transparent PI film (50 μm in thickness) using a laser-cutting system (ultraviolet laser marking machine-3 W, Shenzhen Chaoyue laser Intelligent Equipment Co., Ltd. of China, Shenzhen, China) under 3 W power 3 times (Figure 4a). The laser wavelength was 355 nm; the pulse width was 1 μs; and the laser cutting rates were set to 10 mm/s, 30 mm/s, 40 mm/s and 50 mm/s. Then, a monolayer of double-sided tape (3M 93015LE-300LSE) was cut into a predetermined shape by using the laser-cutting technology, and this operation was repeated three times to form a sweat flow layer with enough height for sweat inflow. After laminating the well-fabricated spiracle layer onto the sweat flow layer, the sample was encapsulated with a single-sided sticky biaxially oriented polypropylene (BOPP) film as the top layer.

During the alignment of the different layers, the double-sided tape (0.15 mm) was first fixed on an operating table to ensure that its position remained unchanged in the carving process. After carving the first layer of tape, the second layer and third layer of double-sided tape were covered for in situ engraving. The position of the double-sided tape did not change during the carving process to ensure that each layer of the structure was aligned. After preparing the sweat flow layer, the spiracle layer (PI film) was manually aligned and adhered to the sweat flow layer with the help of an optical microscope. Because the transparent PI film had a good shape recovery ability, it did not produce deformation during preparation. Moreover, the total thickness of the microfluidic patch was about 0.85 mm.

For the sensing module in the microfluidic patch, we chose the colorimetric method to detect the glucose concentration in sweat. The colorimetric method is based on the color response of the testing sample to a reactant, or a substrate can usually be used to ensure the type of analyte and content or concentration by comparing the color before and after the reaction. Pre-prepared color markers (indicator plates) were put into the four microchambers in the sweat flow layer and encapsulated with a transparent PI film with a hole of 5 mm as the bottom layer. To compensate for the color error caused by varying light intensity, a standard color marker was pasted onto the surface of the top layer. Finally, the microfluidic patch was easily stuck onto skin via double-sided tape with a hole of 5 mm as the adhesive layer.

### 2.3. Experimental Characterization

The morphologies of the CBVs and microchannels in the spiracle layer and sweat flow layer were observed using an optical microscope, a scanning electron microscope (SEM, S-4800 Carl Zeiss SMT Pte. Ltd., Guangzhou, China) and a laser microscope (VK-X110, KEYENCE, Shanghai, China). For the chronological collection test of liquid in the microfluidic patch (in vitro experiment), red dye was uniformly injected into the microchannels with an injection pump (LSP01-3A, Baoding Lange constant flow pump Co., Ltd., China, Baoding, China) at an injection rate of 1 μm/mm. For an in situ experiment, the patch was attached to the forearm of a volunteer exposed to a high temperature while cycling to collect the sweat produced during the exercise.

The glucose concentration in the sweat was examined by using the glucose oxidase (GOD) coupling method. For a colorimetric analysis, a phenol reagent (POD) was mixed with GOD (1:1) to make a GOD-POD solution. Dust-free paper was cut into circular patches with a radius of 2.8 mm, ensuring that the dust-free paper could be embedded into the microreservoir. The dust-free paper was kept soaked in 4 °C GOD-POD solution for 3 h with light avoidance, and the surface was air-dried to form color markers. The color marker could maintain stability at 4 °C for at least 20 days.

The largest color block was selected from the website of Color Hunter, and the software of Color Picker was used to obtain the RGB values of the largest color block; these values were compared with the values of a standard color card to indicate the glucose concentration in the sweat. Each set of colors was taken six times to reduce the error. Subsequently, we prepared different concentrations of the glucose solution and injected them into the microfluidic patch at a uniform speed (1 μm/mm) with an injection pump, resulting in the color change of the patch. In order to investigate the influence of the light of the detection environment on the color of the patch, we placed the patch under three lights with different light intensities (2.5 W white light, 5 W white light and 3 W yellow light) and determined the color value with a color picker and compared it with the standard color card. For selectivity and anti-interference performance tests of the microfluidic patch, a 0.3 mM glucose solution, with the addition of chloride, lactic acid, alcohol and urea, was injected into the patch, and the color values were collected.

For an in situ experiment of sweat glucose detection, we attached the patches to the forearms and chests of two volunteers exposed to a high temperature of 30 °C. At higher temperatures, the volunteers were more likely to sweat while cycling, allowing for sweat to be collected more quickly. All tests of the sweat glucose concentration for each volunteer were carried out by pasting six patches onto the test areas, and the standard deviation was calculated for statistical analyses. During the on-body tests of the sweat glucose concentration for each volunteer, we also measured blood glucose with a commercial blood glucose meter (Yuwell 590, Shanghai, China).

## 3. Results

### 3.1. Morphology of Microfluidic Patch

Figure 4b shows the assembled microfluidic patch, which presents a good flexibility and has a thickness of less than 1 mm (Figure 4c,d), indicating its compatibility with human skin and application potential in the field of sweat management and physiological information monitoring in situ.

**Figure 5:**
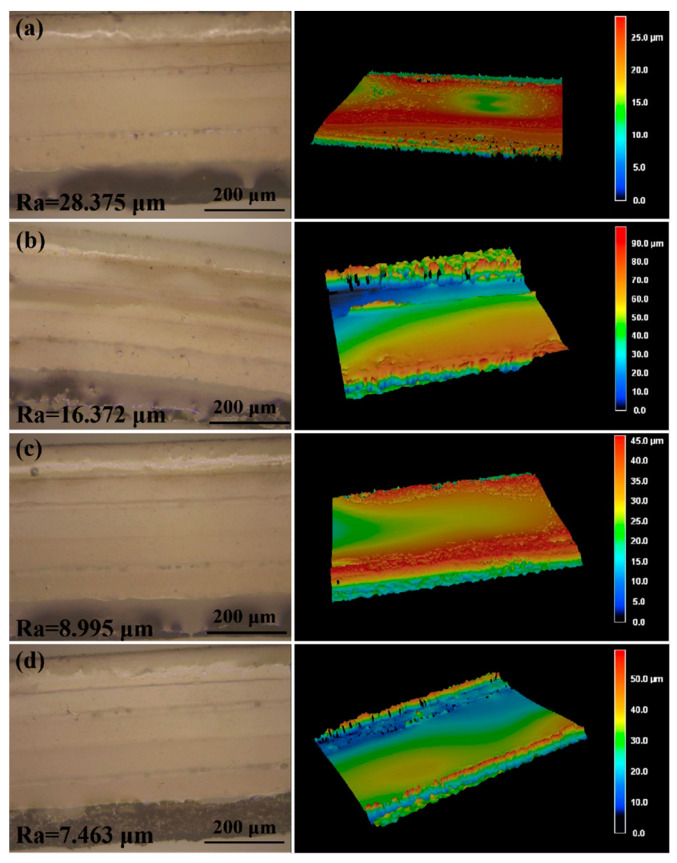
The laser scanning microscope images and the three-dimensional surface topography of the microchannels prepared at different laser cutting rates: (**a**) 10 mm/s, (**b**) 30 mm/s, (**c**) 40 mm/s and (**d**) 50 mm/s. The total processing time was similar.

To obtain the smallest channel size using laser-cutting technology, we adjusted the preset path of the laser to change the width of the channel. We made separate 0.6 mm, 0.4 mm, 0.3 mm, 0.25 mm and 0.2 mm wide channels in the double-sided tape and observed the morphology of the channels by using an optical microscope. When the width of the channel was 0.2 mm, the channel edges had obvious defects (Figure S1a). Similarly, we made separate 0.4 mm, 0.3 mm, 0.25 mm, 0.2 mm, and 0.15 mm wide channels in the transparent PI film, and a well-defined channel was obtained when the width was no less than 0.25 mm (Figure S1b). Therefore, 0.25 mm was the smallest channel size that could be obtained by using laser-cutting technology. When liquid flows into the microchannels in the sweat flow layer, the roughness of the inner walls of the microchannels will influence the sweat flow process. Therefore, we adopted different laser cutting rates (10 mm/s, 30 mm/s, 40 mm/s and 50 mm/s; the total processing time was similar) to obtain microchannels with different roughness. As shown in Figure 5, when the cutting rate increased, the smoothness of the inner wall decreased (from 28.375 μm to 16.372 μm, 8.995 μm and 7.463 μm) because slower laser processing can cause more burnt inner walls [20,21]. Therefore, the optimized parameter of laser treatment for obtaining smooth inner walls in the microchannels was 50 mm/s. The detection results of sweat collection indicate that the roughness of the microchannel inner wall would have a certain effect on the collection time of sweat (Figure S2).

### 3.2. Chronological Collection of Liquid in the Microfluidic Patch

**Figure 6:**
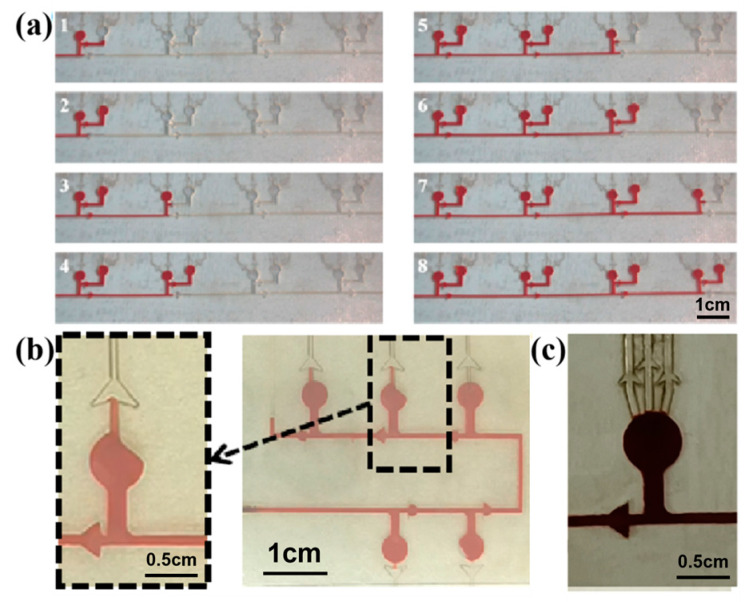
(**a**) CBV#1 takes effect when the secondary microreservoirs are set up. (**b**) When setting a single spiracle valve, part of the air is blocked in the microreservoir and cannot be released. (**c**) Three parallel CBV#2 vaults are designed to ensure that the air can be vented out thoroughly.

In order to realize the chronological collection of liquid in the microfluidic patch, two different types of CBVs (CBV#1 and CBV#2) are designed. Then, red dye is uniformly injected into the microchannels with an injection pump at an injection rate of 1 μm/mm. When the red dye liquid enters the microreservoir, it flows into the next set of microreservoirs after filling the secondary branch, as shown in Figure 6a, indicating the effectiveness of CBV#1. However, in some cases, the liquid preferentially fills one side of the microreservoir, and sweat blocks the spiracle valve (CBV#2), as shown in Figure 6b, so the residual air in the microreservoir cannot be vented out; therefore, sweat will not fill the microreservoir. To solve this problem, we design three parallel spiracle valves (CBV#2), ensuring that the air can be vented out thoroughly, as shown in Figure 6c.

**Figure 7:**
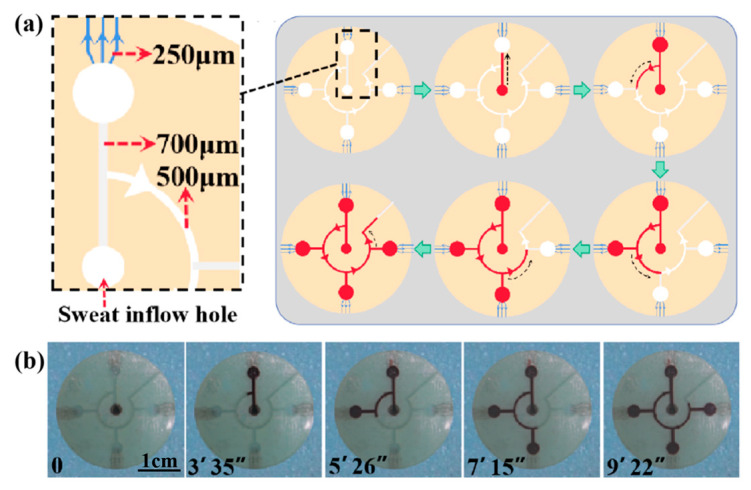
(**a**) A schematic diagram of the entire platform for chronological collection of liquid. (**b**) The time took for the liquid to fill each microreservoir.

To verify the effect of the CBVs (CBV#1 and CBV#2) in the microfluidic patch for sweat collection, we injected red dye into the patch via an injection pump at an injection rate of 1 μm/mm (Video S1). Figure 7a shows a simulation diagram of sweat flowing into the four microreservoirs successively under the BP of CBV#1. As shown in Figure 7b, the time it takes for the dye to fill the entire patch is about 9 min and 22 s, and it fills each microreservoir in sequence for about 2 min. Therefore, our results indicate that CBV#1 can control the flow of solution in sequence, and CBV#2 can ensure that the liquid does not leak out.

### 3.3. Colorimetric Measure for Glucose Concentration in Sweat

**Figure 8:**
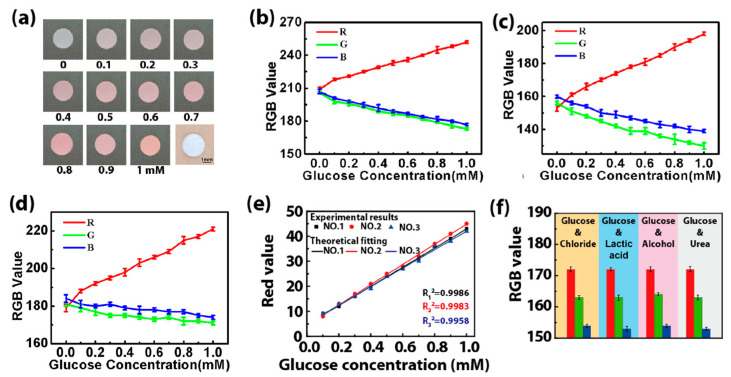
(**a**) Pictures of the color markers with different concentrations of glucose solution (0.1–1 mM). (**b**–**d**) The RGB value when the color markers are illuminated with different kinds of light: (**b**) 2.5 W white light, (**c**) 5 W white light and (**d**) 3 W yellow light. (**e**) The final reference curves obtained by fitting the R values of the color markers with different concentrations of the glucose solution under different kinds of light irradiation (no. 1: 2.5 W white light, no. 2: 5 W white light, no. 3: 3 W yellow light). (**f**) The RGB values of color markers when chloride (0.3 mM), lactic acid (0.3 mM), alcohol (0.3 mM) and urea (0.3 mM) were added into the glucose solution (0.3 mM). The standard deviation was calculated from six test results at the test areas for each volunteer.

The colorimetric method is selected to detect the glucose concentration in sweat via an enzyme-based reagent [22]. Catalyzed by GOD, glucose is oxidized to gluconic acid, which couples with oxygen to produce H_2_O_2_. Then, the produced H_2_O_2_ oxidizes the colorless o-dianisidine to generate red-colored oxidized o-dianisidine. The presence of glucose brings out the red color of the GOD-peroxidase-o-dianisidine system [23]. Therefore, the amount of H_2_O_2_ generated in the initial reaction is directly proportional to the glucose concentration [24]. The color markers consisting of GOD and POD in the microreservoir have a sensitive color reaction to H_2_O_2_ with different concentrations, presenting an obvious color change related to glucose concentration. Figure 8a shows images of color markers with different concentrations of the glucose solution dropped onto them, presenting large differences in the color value. This indicates that the color markers can distinguish and determine the concentration of glucose in sweat with a color picker. When the color markers with different concentrations of the glucose solution (0.1–1 mM) are placed under three different kinds of light (2.5 W white light, 5 W white light and 3 W yellow light), the RGB value of the color response to glucose is found to be linear with the glucose concentration for these three different kinds of light (Figure 8b–d). The linear fitting of their RGB values shows that the R value has a high fitting degree (Figure 8e), which can be used as the final reference curve for the colorimetric result [25,26]. We also study the selectivity of the color markers for glucose by performing four sets of contrast tests in which chloride, lactic acid, alcohol and urea solutions with the same concentrations (0.3 mM) were added to a 0.3 mM glucose solution. The RGB value is almost constant (Figure 8f), proving that the color marker is highly selective for glucose, and it can effectively prevent interference from other biomarkers in sweat.

**Figure 9:**
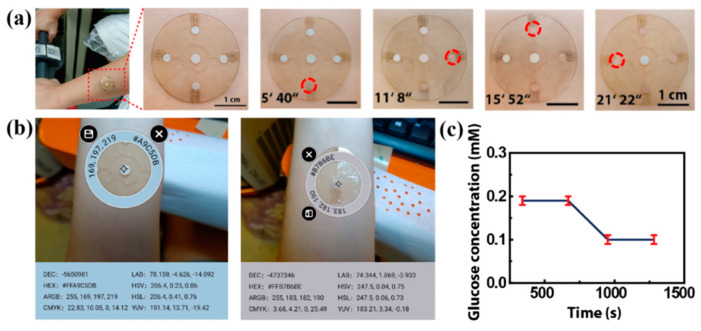
The on-body experiments: (**a**) microfluidics patch attached to the forearm of a volunteer. The time of sweat flowing into each microreservoir is shown, and the sweat makes the color marker allochroic. (**b**) A physical picture of the microfluidic patch on a volunteer’s forearm. The RGB values of the standard color card and the colored color markers are obtained with a color picker. (**c**) The glucose concentration in the volunteer’s sweat, referring to the fitting line in Figure 8e.

For an in situ experiment, we attached the patch to the forearm of a volunteer exposed to a high temperature of 30 °C (Figure 9a,b). When they cycle, sweat flows from the center hole and into the microreservoirs. After about 5 min, the sweat fills the first microreservoir and makes the color change (Figure 9c). The time it takes for the sweat to fill each microreservoir is shown in Figure 9c, with the total time being about 21 min and 22 s. The RGB values of the color markers in the four microreservoirs are obtained by using a color picker, and they are contrasted with the standard color card in the center. Referring to the fitting line in Figure 8e, the glucose concentration in the volunteer’s sweat is determined to be about 0.19 mM after 0–12 min of cycling, and it decreases to 0.10 mM after 15–21 min of cycling (Figure 9c) due to the glucose loss caused by movement. This result is consistent with that reported in previous work [23].

When we attach two sets of patches to the forearm and chest of one volunteer (Volunteer 1), the sweat collection from the forearm (black line) is slower than that from the chest (red line) due to the lower sweat rate of the forearm (Figure S3a). However, the glucose concentrations are essentially the same at the same times. This indicates that, for the same individual, different parts of the skin would not affect the function of the patch. For different individuals with different densities of sweat glands, for example, wider or thinner arms, and hairy or hairless skin, the sweat rate has individual variation, but the change trend of sweat glucose is basically the same. As shown in Figure S3, usually, hairy skin (with more sweat glands) has a higher rate of sweat collection than hairless skin. Although sweat collection is faster on the chest than on the forearm, the glucose concentrations are the same basically at the same times; that is, the sweat rate does not affect the glucose concentration. Additionally, because the valves in the patch are not in direct contact with the skin, body hair will not affect the air flow into the patch, and the liquid circulation is normally not blocked. So, hairy or hairless state does not affect the detection result. During the on-body tests of the sweat glucose concentration for each volunteer, we also measured blood glucose with a commercial blood glucose meter (Yuwell 590). The change trend of blood glucose detected by using the commercial blood glucose meter is basically the same as that obtained by using our microfluidic patch (Figure S3). There is no inconvenience or restriction for the volunteer during exercise, no discomfort or irritation on their skin surface and no reduced adhesion or fluid leakage during collection, indicating the favourable usability of the microfluidic patch for the detection of glucose concentration in sweat.

Of course, there are still some limitations to our microfluidic patch. Firstly, compared with soft-lithography technology, laser-cutting technology has a lower fineness. Through the structural design of the CBVs, the effective collection and transportation of sweat can be achieved. Secondly, the inner wall of the microchannel obtained by using the laser-cutting method is rough to a certain extent, which has a certain effect on the collection time of sweat but almost has no effect on the detection of the sweat glucose concentration (Figure S2). Finally, different light intensities may affect the measurement result of RGB values, although we minimized the error by setting a standard color card. Multiple measurements are sensible to determine the true concentration.

## 4. Conclusions

In summary, we proposed a microfluidic patch fabricated using a low-cost, simple laser-cutting manufacturing technique. The microfluidic patch has the functions of collecting, storing and detecting sweat in chronological order via the manipulation of different types of CBVs. Moreover, the microfluidic patch can detect the glucose concentration in sweat effectively with a high selectivity and an excellent anti-interference ability, indicating its application potential in sweat management and the physiological information monitoring of humans.

## Data Availability

All relevant data are included in the manuscript and Supplementary Materials.

## Supplementary Materials

The following supporting information can be downloaded at https://www.mdpi.com/article/10.3390/bios13030372/s1, Figure S1. (a) The 0.6 mm, 0.4 mm, 0.3 mm, 0.25 mm, and 0.2 mm-wide channels made in the double-side tape, and (b) the 0.4 mm, 0.3 mm, 0.25 mm, 0.2 mm, and 0.15 mm-wide channels made in the transparent PI film by the laser-cutting technology; Figure S2. The glucose concentration detected by using the microfluidic patches with various roughness; Figure S3. On-body experiments of (a) the hairy volunteer (Volunteer 1), and (b) the hairless vol-unteer (Volunteer 2).Video S1: The entire platform for chronological collection of liquid.
