# Supplementary material for: Laser-Cutted Epidermal Microfluidic Patch with Capillary Bursting Valves for Chronological Capture, Storage, and Colorimetric Sensing of Sweat
Source: Biosensors (Basel). 2023 Mar 12;13(3):372. doi: 10.3390/bios13030372 (PMC10046219; doi:10.3390/bios13030372)
Supplement: Supplementary file 1 [file biosensors-13-00372-s001.zip › biosensors-2242633-Supporting information.pdf]

biosensors

Supporting information

Laser-Cutting of Epidermal Microfluidic Patch with Capillary Bursting Valves for Chronological Capture, Storage and Colorimetric Sensing of Sweat

Yuxin He [1,†], Lei Wei [1,2,†], Wenjie Xu [1], Huaping Wu [3] and Aiping Liu [1,*]

* Correspondence: liuaiping1979@gmail.com
† These authors contributed equally to this work.

Abstract: Flexible wearable microfluidic devices show great feasibility and potential development in the collection and analysis of sweat due to their convenience and non-invasive characteristics in health-level feedback and disease prediction. However, the traditional production process of microfluidic patches relies on resource-intensive laboratory and high-cost facilities. In this paper, a low-cost laser-cutting technology is proposed to fabricate epidermal microfluidic patches for the collection, storage and colorimetric analysis of sweat. Two different types of capillary bursting valves are designed and integrated into microchannel layers to produce two-stage bursting pressure for the reliable routing of sweat into microreservoirs in sequential fashion, avoiding the mixing of old and new sweat. Additionally, an enzyme-based reagent is embedded into the microreservoirs to quantify the glucose level in sweat by using colorimetric methods, demonstrating a high detection sensitivity at the glucose concentration from 0.1 mM to 1 mM in sweat and an excellent anti-interference performance that prevents interference from substances probably existent in sweat. In vitro and on-body experiments demonstrate the validity of the low-cost, laser-cut epidermal microfluidic patch for the chronological analysis of sweat glucose concentration and its potential application in the monitoring of human physiological information.

Keywords: laser-cutting technology; microfluidics; capillary bursting valves; glucose level; colorimetric sensing

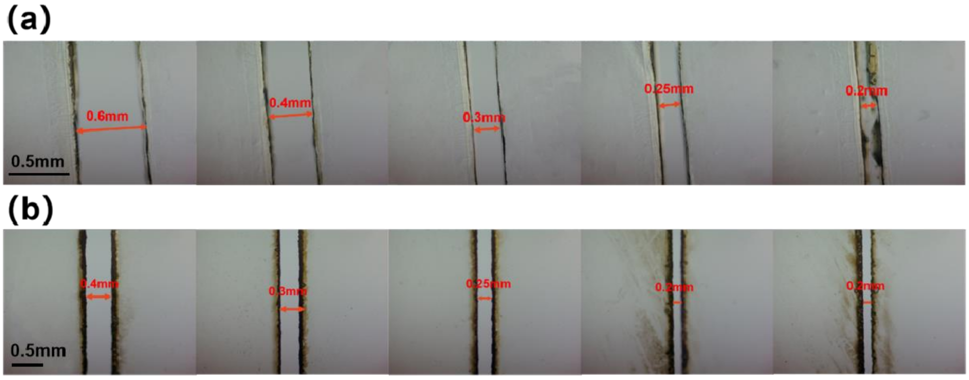

Figure S1. (a) The 0.6 mm, 0.4 mm, 0.3 mm, 0.25 mm, and 0.2 mm-wide channels made in the double-side tape, and (b) the 0.4 mm, 0.3 mm, 0.25 mm, 0.2 mm, and 0.15 mm-wide channels made in the transparent PI film by the laser-cutting technology.

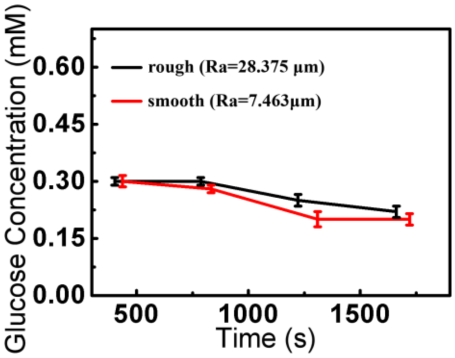

Figure S2. The glucose concentration detected by using the microfluidic patches with various roughness.

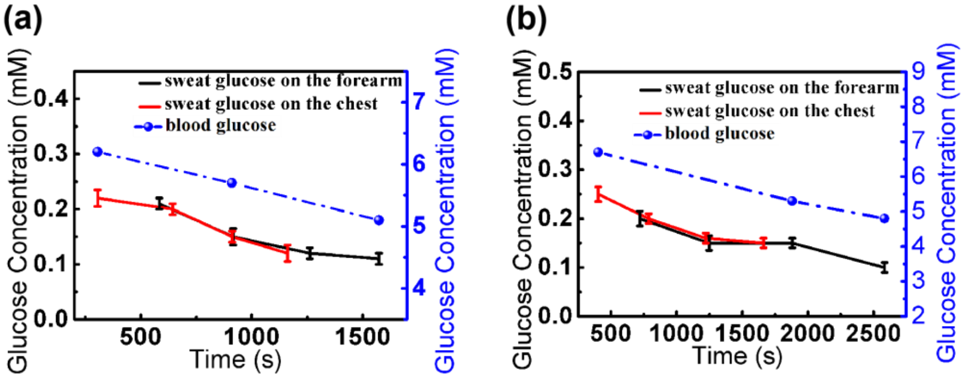

Figure S3. On-body experiments of (a) the hairy volunteer (Volunteer 1), and (b) the hairless volunteer (Volunteer 2).
